# Mutation of the Enterohemorrhagic *Escherichia coli* Core LPS Biosynthesis Enzyme RfaD Confers Hypersusceptibility to Host Intestinal Innate Immunity *In vivo*

**DOI:** 10.3389/fcimb.2016.00082

**Published:** 2016-08-12

**Authors:** Cheng-Ju Kuo, Jenn-Wei Chen, Hao-Chieh Chiu, Ching-Hao Teng, Tai-I Hsu, Pei-Jung Lu, Wan-Jr Syu, Sin-Tian Wang, Ting-Chen Chou, Chang-Shi Chen

**Affiliations:** ^1^Institute of Basic Medical Sciences, College of Medicine, National Cheng Kung UniversityTainan, Taiwan; ^2^Department of Biochemistry and Molecular Biology, College of Medicine, National Cheng Kung UniversityTainan, Taiwan; ^3^Center of Infectious Disease and Signaling Research, National Cheng Kung UniversityTainan, Taiwan; ^4^Department of Clinical Laboratory Sciences and Medical Biotechnology, National Taiwan UniversityTaipei, Taiwan; ^5^Institute of Molecular Medicine, College of Medicine, National Cheng Kung UniversityTainan, Taiwan; ^6^Institute of Clinical Medicine, College of Medicine, National Cheng Kung UniversityTainan, Taiwan; ^7^Institute of Microbiology and Immunology, National Yang Ming UniversityTaipei, Taiwan

**Keywords:** enterohemorrhagic *Escherichia coli* (EHEC), RfaD/GmhD/WaaD, lipopolysaccharide (LPS), antimicrobial peptides (AMPs), intestinal innate immunity, *Caenorhabditis elegans*

## Abstract

Enterohemorrhagic *Escherichia coli* (EHEC) O157:H7 is an important foodborne pathogen causing severe diseases in humans worldwide. Currently, there is no specific treatment available for EHEC infection and the use of conventional antibiotics is contraindicated. Therefore, identification of potential therapeutic targets and development of effective measures to control and treat EHEC infection are needed. Lipopolysaccharides (LPS) are surface glycolipids found on the outer membrane of gram-negative bacteria, including EHEC, and LPS biosynthesis has long been considered as potential anti-bacterial target. Here, we demonstrated that the EHEC *rfaD* gene that functions in the biosynthesis of the LPS inner core is required for the intestinal colonization and pathogenesis of EHEC *in vivo*. Disruption of the EHEC *rfaD* confers attenuated toxicity in *Caenorhabditis elegans* and less bacterial colonization in the intestine of *C. elegans* and mouse. Moreover, *rfaD* is also involved in the control of susceptibility of EHEC to antimicrobial peptides and host intestinal immunity. It is worth noting that *rfaD* mutation did not interfere with the growth kinetics when compared to the wild-type EHEC cells. Taken together, we demonstrated that mutations of the EHEC *rfaD* confer hypersusceptibility to host intestinal innate immunity *in vivo*, and suggested that targeting the RfaD or the core LPS synthesis pathway may provide alternative therapeutic regimens for EHEC infection.

## Introduction

Enterohemorrhagic *Escherichia coli* (EHEC), the most recognized serotype of which is O157:H7, is a gram-negative foodborne pathogen which can cause diarrhea and other severe symptoms, like hemorrhagic colitis (HC), hemolytic-uremic syndrome (HUS) and acute renal failure in humans (Pennington, 2010). However, treatment of EHEC infection with antibiotics is not recommended because more EHEC virulence factors, such as Shiga toxins and endotoxins, may be released and exacerbate severe syndromes in patients (Pacheco and Sperandio, 2012; Freedman et al., 2016). Currently, only palliative care can be applied after EHEC infection. Therefore, the development of a novel therapeutic strategy for EHEC infection is urgently needed. Several virulence factors expressed by EHEC contribute to its pathogenicity (Nguyen and Sperandio, 2012). The Shiga toxins (Stxs) produced by EHEC are responsible for HUS and HC. The type III secretion system (T3SS) and effectors encoded by the locus of enterocyte effacement (LEE) pathogenicity island are required for formation of the attaching and effacing (A/E) lesions on host intestinal epithelium. Although many virulence factors and mechanisms of EHEC have been reported, therapeutic strategies targeting these specific virulence factors remain under investigation (Nguyen and Sperandio, 2012; Pacheco and Sperandio, 2012).

**Figure 1:**
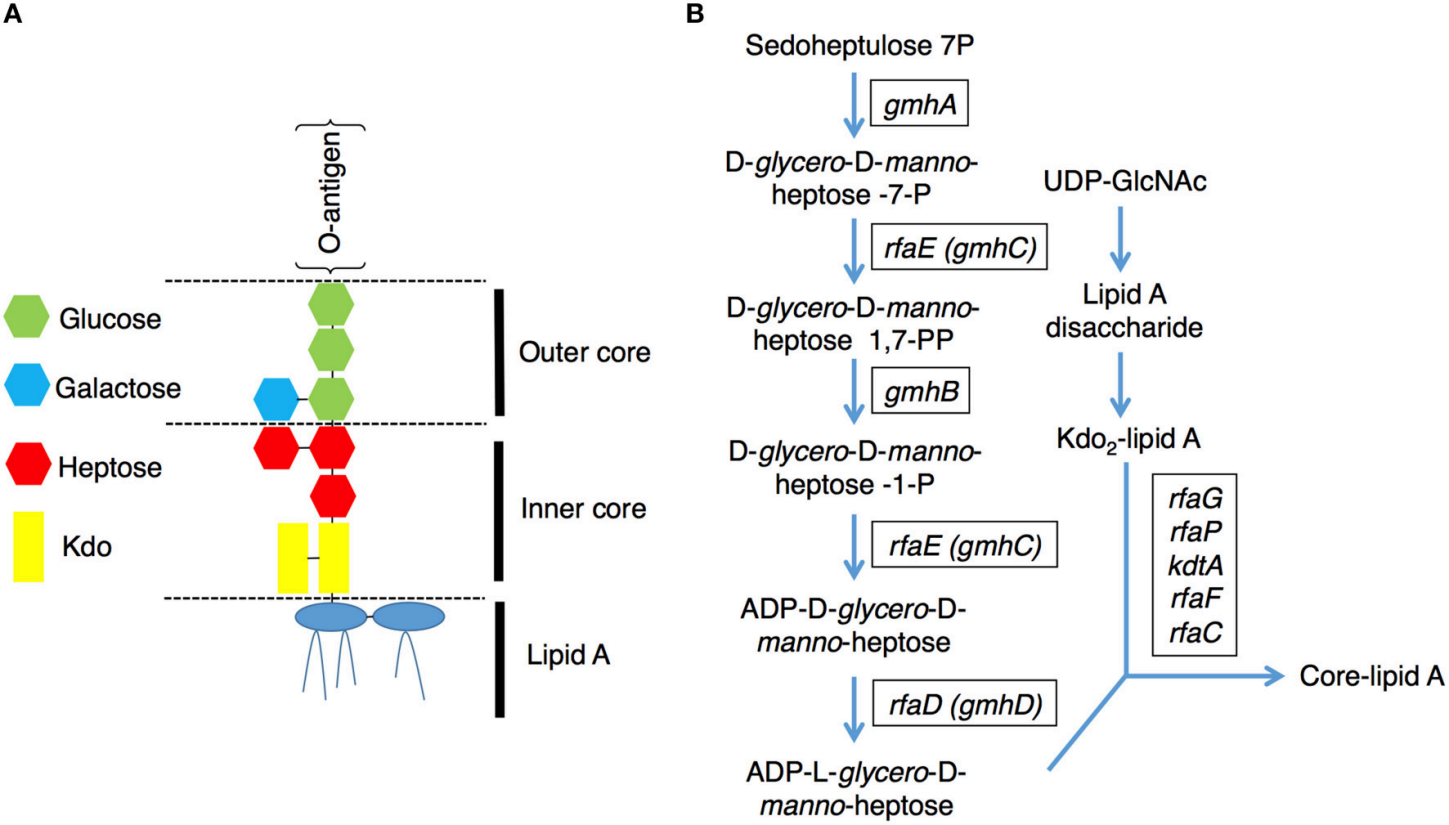
**Lipopolysaccharide (LPS) structure and the core-lipid A biosynthetic pathway**. **(A)** A graphic representation of the LPS structure of *E. coli*. LPS contains several conserved components: lipid A, inner core, outer core and O-antigen (Raetz and Whitfield, 2002). **(B)** Pathways for the biosynthesis of the LPS core lipid A (Raetz and Whitfield, 2002). The genes encode the catalytic enzymes for ADP-L-*glycero*-D-*manno*-heptose and core-lipid A biosynthesis are presented in rectangular boxes.

Lipopolysaccharides (LPS) are surface phosphorylated lipoglycans on the outer leaflet of the outer membrane in gram-negative bacteria (Raetz and Whitfield, 2002), including EHEC. *E. coli* LPS is a complex molecule that can be divided structurally into three parts (Figure 1A), lipid A, core oligosaccharides, and O-antigen polysaccharide chains (Raetz and Whitfield, 2002). The core oligosaccharide can be further divided into the inner and outer cores respectively. The inner core is composed of 3-deoxy-D-*manno*-oct-2-ulosonic acid (Kdo) and ADP-heptose residues, and the outer core provides the attachment site for the O-polysaccharide chain and is constructed of hexoses and 2-acetoamido-2-deoxy-hexoses. ADP-heptose is a crucial component of the LPS inner core, and it connects the outer part of LPS to Kdo between the Kdo_2_-lipid A and O-antigen. Moreover, ADP-heptose is also required to maintain outer membrane integrity, restrict permeability and prevent attack by the complement system (Raetz and Whitfield, 2002; Chang et al., 2011). The *E. coli* chromosomal *rfa*, also known as *gmh* or *waa*, operons encode enzymes needed for the stepwise assembly of the major core oligosaccharides (Raetz and Whitfield, 2002). The biosynthetic pathway for the nucleotide-activated *glycero*-*manno*-heptose precursors of LPS has been characterized in *E. coli* (Figure 1B). It contains five catalytic steps that are required for generation of ADP-L-*glycero*-D-*manno*-heptose, a molecule essential for the first heptose and can be transferred to Kdo (Valvano et al., 2002). The *rfaD* gene, also named *gmhD* or *waaD*, encodes the ADP-L-*glycero*-D-*manno*-heptose-6-epimerase which catalyzes the conversion of ADP-D-*glycero*-D-*manno*-heptose to ADP-L-*glycero*-D-*manno*-heptose, the last step in the ADP-L-*glycero*-D- *manno*-heptose synthetic pathway. The biochemistry of this biosynthetic pathway in *E. coli* has long been characterized; however, targeting RfaD and the core LPS biosynthesis enzymes for the treatment of EHEC infection remains largely unexplored.

Many animal models have been developed to facilitate the study of EHEC pathogenesis. Such models have been used to explore differences in relative toxicity among different types of Stxs (Tesh et al., 1993), assess the protective capacity of novel therapeutic methods (Sheoran et al., 2005; Armstrong et al., 2006; Bentancor et al., 2009), and model the pathogenesis of HUS (Sauter et al., 2008). Here, we utilized the model organism *Caenorhabditis elegans*, which might encounter EHEC in its natural habitat, as a surrogate host to study EHEC infection (Kenney et al., 2005; Anderson et al., 2006; Chou et al., 2013). Several characteristics of *C. elegans* make it suitable for studying pathogen and host interactions, including its small size, rapid life cycle, ease of conducting forward, reverse and chemical genetic screens, and sharing of conserved innate immune pathways with humans (Irazoqui et al., 2010), which are invaluable for investigating infection. Of particular relevance, *C. elegans* intestinal cells share similar anatomic features with humans (McGhee, 2007), which makes it an attractive model for the study of intestinal pathogens, including EHEC. We have demonstrated that EHEC can colonize and induce characteristic A/E lesions in the intestine of *C. elegans*, and Shiga toxin 1 (Stx1) is also required for the full virulence of EHEC pathogenicity in *C. elegans* (Chou et al., 2013). All these facts suggest that EHEC exerts similar and conserved virulence mechanisms in *C. elegans* and humans.

Herein, we report that inactivation of the EHEC RfaD and other core LPS biosynthesis enzymes attenuated its toxicity to *C. elegans.* Moreover, deletion of the EHEC *rfaD* gene significantly reduced its intestinal colonization in animal hosts, including *C. elegans* and mouse *in vivo*, and increased its susceptibility to several antimicrobial peptides (AMPs) *in vitro*. Our results highlight the importance of RfaD and core LPS in host intestinal colonization and suggest targeting RfaD or the core LPS synthesis pathway in EHEC may provide as potential therapeutic strategies for EHEC infection.

## Materials and methods

### Bacterial and *C. elegans* strains

The bacterial strains and plasmids used for this study are listed in Tables S1, S2 respectively. The enterohemorrhagic *E. coli* O157:H7 clinical isolates, EDL933 and HER1266, were from the Bioresource Collection and Research Center (BCRC, Taiwan). The EHEC mutants were generated by the lambda Red recombinase system (Datsenko and Wanner, 2000) and described in the Supplementary Information. All EHE-related biohazardous wastes were disinfected and disposed according to the Biosafety Level 2 (BSL-2) regulation. The Bristol N2 is the wild-type *Caenorhabditis elegans* strain. The DA597 strain contains two mutations in the *phm-2(ad597)* allele and results in abnormal function of the pharynx (Avery, 1993). The GK454 strain with the mCherry::ACT-5 transgene was used to monitor the microvillar actin rearrangement (Sato et al., 2011; Chou et al., 2013). *C. elegans* strains were maintained on nematode growth medium (NGM) agar plates using the standard nonpathogenic *E. coli* strain OP50 and synchronized with alkaline hypochlorite solution as described (Brenner, 1974).

### Survival analysis

All survival assays were conducted as previously described (Chou et al., 2013). In brief, EHEC was cultured in Luria-Bertani (LB) broth overnight at 37°C. Thirty to fifty synchronized late L4 to young adult stage *C. elegans* animals were transferred to each NGM agar plate, which was spread with the 30 μl bacterial overnight cultures and kept at 20°C. Animals were monitored daily and transferred to fresh plates daily until no more progeny were produced. Animals that did not respond to gentle prodding by platinum wire and displayed no pharyngeal pumping were scored as dead. Survival was monitored over time until all animals had died. The experiment was performed independently three times with approximately 100 animals per EHEC strain each time. Survival analysis was performed using GraphPad Prism 5.0 (GraphPad Software, La Jolla, CA). The Mantel-Cox logrank test was used to assess statistical significance of difference in survival, and *P* < 0.05 were considered significant.

### LPS extraction and visualization

Lipopolysaccharides of EHEC were prepared by a hot phenol–water method as described (Chang et al., 2011), resolved on 12% SDS–polyacrylamide gels and visualized by silver staining as described previously (Yan et al., 2000).

### Bacterial colonization

Experimental procedures were performed as described (Chou et al., 2013). For the Green fluorescent protein (GFP) image analysis, synchronized late L4 to young adult stage *C. elegans* animals (N2 or DA597) were fed with either GFP-labeled *E*. *coli* OP50, *E*. *coli* O157:H7 EDL933, EDL933:Δ*rfaD* or EDL933:Δ*rfaD-prfaD* bacteria for 1 day at 20°C and then transferred to non GFP-labeled *E*. *coli* OP50 bacterial plates for another 3 days. The infected animals were collected and mounted on glass slides with 2% agarose pads and anesthetized with 25 nM sodium azide (NaN_3_). Colonization of the GFP-labeled bacteria in the animals was imaged by a Nikon Eclipse Ti inverted microscope system and quantified. For the colony forming unit (CFU) analysis, the infected animals were washed out from the plates, treated with 25 mM levamisole and washed in M9 medium 10 times. To eliminate the bacteria outsite of the animals, the infected animals were then treated with M9 buffer containing 25 mM levamisole, 100 μg/ml gentamicin, and 1mg/ml ampicillin for 1–2 h at room temperature. These antibiotics were eliminated by washing the worms in M9 buffer with 25 mM levamisole 3 times. Ten animals were picked randomly into 100 μl M9 buffer, pulverized for 1 minute using a sterile plastic pestle, and plated on LB agar containing ampicillin after serial dilution. The numbers of bacterial colonies were counted the next day and the colony forming unit (CFU) per animal was calculated.

### Microvillar ACT-5 cellular localization

EHEC induces A/E lesion characterized by the effacement of intestinal microvilli and the rearrangement of actin cytoskeleton in the host intestinal cells. ACT-5 is the main actin isoform that constitutes the intestinal microvilli in *C. elegans* (MacQueen et al., 2005). To test whether EHEC induces intestinal microvillar actin rearrangement, we analyzed distribution of the mCherry-tagged ACT-5 in *C. elegans* as previously described (Chou et al., 2013). In brief, the GK454 animals, with the *dkIs247*(*act-5p::*mCherry::HA*::act-5)* transgene were fed with *E*. *coli* OP50, *E*. *coli* O157:H7 EDL933, EDL933 *rfaD::Tn5*, EDL933:Δ*rfaD*, or EDL933:Δ*rfaD*-*prfaD* for 4 days and the mislocalization of the mCherry::ACT-5 signals from the apical membrane to the cytoplasm of the intestinal cells (ectopic ACT-5 localization) was examined. The infected animals were paralyzed by the method described above and images were captured by Nikon C1-Si confocal microscope.

### Serum killing assay

Experiments followed the procedures previously described (Wang et al., 2012). Briefly, Log-phase bacteria (~10^5^ CFU) were incubated in 100 μl of 10% normal human serum (NHS) or heat-inactivated normal human serum (HNHS) diluted in PBS at 37°C. Aliquots (10 μl) were collected after 60 min, and each sample was spread on to an LB agar plate. After culturing overnight, the bacterial numbers were counted. The survival ratios were calculated by the ratio of the bacterial counts to those of the originally inoculated.

### *in vivo* and *ex vivo* imaging analysis of EHEC infected mice

The experiments were approved by the Institutional Animal Care and Use Committee of National Cheng Kung University (NCKU) (Approval number 102-209 and 104-039), and all experiments were carried out in accordance with the approved guidelines and regulations. Experiments were conducted as described previously with slight modifications (Rhee et al., 2011). In brief, the 6-week-old C57BL/6 female mice were purchased and maintained at the Laboratory Animal Center of NCKU. Mice were supplied with drinking water containing streptomycin sulfate (5 g/L) for 24 h and then switched to regular water for another 24 h before infection. Bacteria transformed with pWF279 (luciferase expressing plasmid) were cultured overnight at 37°C in LB medium and diluted in 1:100 in LB medium for 2.5–3 h at 37°C. Bacteria then were centrifuged and washed with sterile normal saline twice and the bacterial concentration was adjusted to approximately 10^9^ CFU/100 μl in normal saline for mouse oral gavage with a 22 gage, ball-tipped feeding needle. The bacterial concentrations were confirmed by plating on LB agar plates. One hour after oral inoculations, the bioluminescent signals in infected mice were examined by Xenogen IVIS 200 imaging system under anesthesia by inhalation of 2.5% isoflurane with 1.5L/min oxygen. Two days post-infection, the bioluminescent signals in the mice were examined again. The mice were then euthanized by cervical dislocation by trained personnel and the entire intestines of infected mice were removed, positioned on a 9 cm Petri dish and imaged by IVIS *ex vivo.*

### Susceptibility to antimicrobial peptides

LL-37 peptide (LLGDFFRKSKEKIGKEFKRIVQRIKDFL RNLVPRTES) was synthesized by MDBio, Taiwan. The purity (>97%) and molecular weight were confirmed by high performance liquid chromatography and mass spectroscopy. Polymyxin B (catalog number: P0972) and Colistin (catalog number: C4461) were purchased from Sigma-Aldrich. In order to test the susceptibility of EDL933 *rfaD* mutants to AMPs, including LL-37, Polymyxin B and Colistin, we measured the bacterial growth with different dosages of AMPs by the microtiter broth dilution method as described (Giacometti et al., 2000; Thwaite et al., 2006; Wiegand et al., 2008). In brief, 37°C overnight bacterial culture in cation-adjusted Mueller Hinton (MH) medium was diluted in 1:100 in MH medium and further cultured at 37°C for 2.5–3 h. The bacteria in the exponential phase were then adjusted to approximately 10^4^ CFU/100 μl by MH medium. Microtiter plates were prepared with serial concentrations of AMPs, ranging from 1.5625 to 100 μg/ml for LL-37 in MH broth. A suspension containing 100 μl of diluted bacteria (10^4^ CFU) was added to a well of a 96-well microtiter plate containing peptide, and then the plate was incubated at 37°C for 16 h. Bacterial growth was determined by measuring the absorbance at 595 nm with a Multiskan FC microplate photometer (Thermo Scientific), or by the OD-monitor (TAITEC, Japan). The bacterial CFU were confirmed by plating on the LB agar plates.

### Data analysis

All experiments were performed a minimum of three times independently. Statistical analysis between two values was compared with a paired *t*-test, and among three or more values of one independent variable was done with matched one-way ANOVA with Tukey's method and more than two independent variables by two-way ANOVA with the Bonferroni post test. All data analysis was performed using SPSS, ver 13.0 (SPSS, Chicago, IL). Statistical significance was set at *P* < 0.05.

## Results

### Disruptions of the *rfaD* and core LPS biosynthesis genes decrease toxicity of EHEC O157:H7 to *C. elegans*

**Figure 2:**
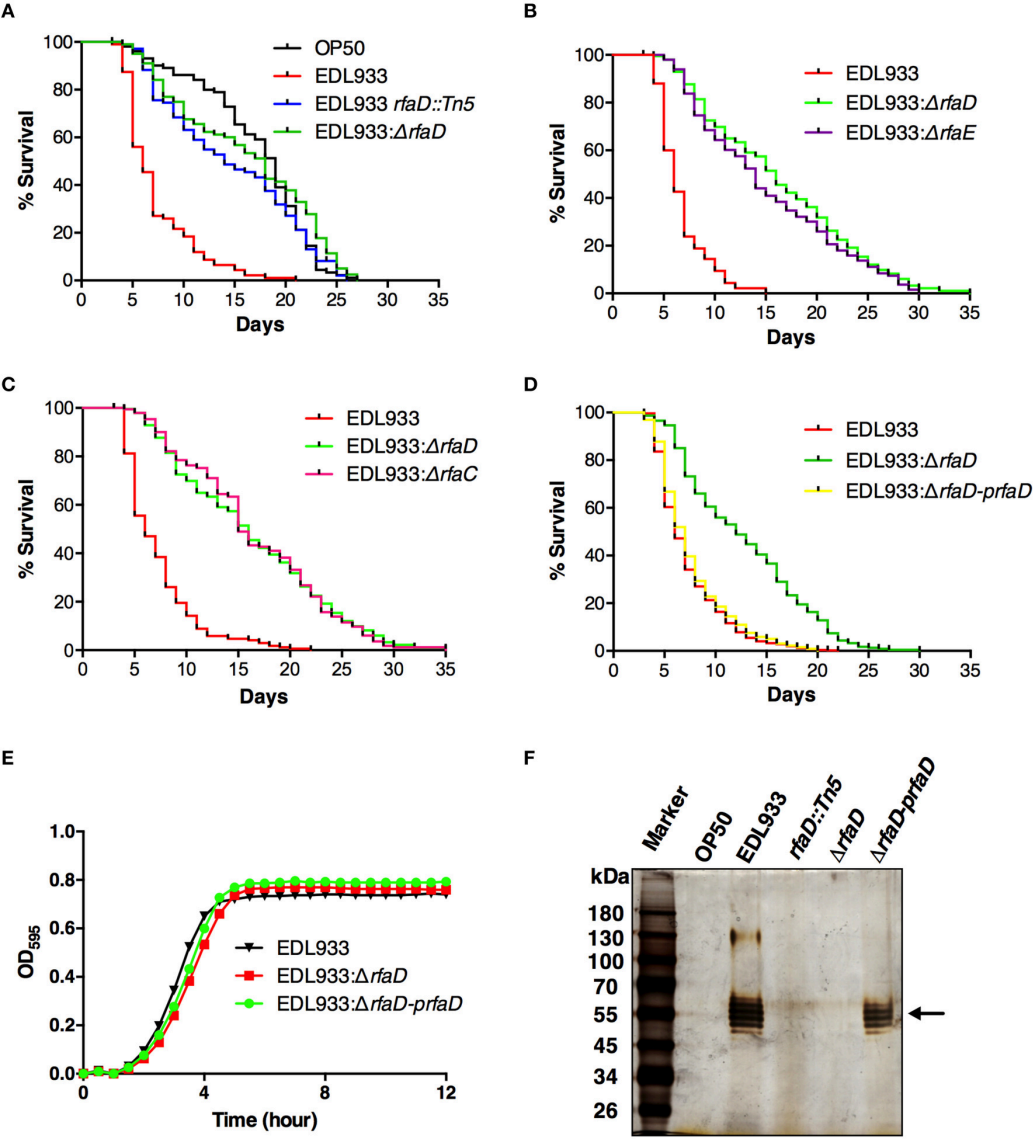
**Disruptions of *rfaD* and the genes involved in EHEC core LPS biosynthesis confer attenuated toxicity. (A–D)** Survival curves of N2 animals fed with *E. coli* strain OP50 (OP50), EHEC strain EDL933 (EDL933), and EDL933 with Tn5 transposon insertion in *rfaD* (EDL933 *rfaD::Tn5*), *rfaD* deletion (EDL933:Δ*rfaD*), *rfaE deletion* (EDL933:Δ*rfaE*) *and rfaC deletion* (EDL933:Δ*rfaC*), and EDL933:Δ*rfaD* with *prfaD* complementation (EDL933:Δ*rfaD-prfaD*) were examined. All survival experiments were conducted independently at least three times with approximately 100 animals each time. **(A)** Animals feeding on the EDL933 *rfaD::Tn5* mutant plates (*P* < 0.001) and the EDL933:Δ*rfaD* plates (*P* < 0.001) lived significantly longer than animals feeding on the wild-type EDL933 plates. **(B)** N2 animals fed with EDL933*:*Δ*rfaE* (*P* < 0.001) lived significantly longer than animals fed with the wild-type EDL933, but were similar to those fed with EDL933*:*Δ*rfaD* (*P* = 0.100). **(C)** Animals feeding on EDL933*:*Δ*rfaC* plates (*P* < 0.001) lived significantly longer than animals feeding on the wild-type EDL933 plates, but were similar to those on EDL933*:*Δ*rfaD* (*P* = 0.938). **(D)** N2 animals fed with EDL933:Δ*rfaD* (*P* < 0.001) lived significantly longer than animals fed with EDL933 wild type. The survival curve of N2 animals fed with EDL933:Δ*rfaD-prfaD* was similar to that of wild-type EDL933 (*P* = 0.128). **(E)** Growth curves of wild-type EDL933 and *rfaD* mutants in LB broth at 37°C. Both the growth kinetic curves of EDL933:Δ*rfaD* and EDL933:Δ*rfaD-prfaD*, were similar to wild-type EDL933. **(F)** The LPS samples of OP50, EDL933, EDL933 *rfaD::Tn5*, EDL933:Δ*rfaD*, and EDL933:Δ*rfaD-prfaD* was examined by silver staining. O-antigens are indicated by the black arrow.

LPS has been reported to play important roles in the pathogenesis of many pathogenic bacteria, including EHEC (Miyashita et al., 2012). From a genetic screen for identification of attenuated EHEC mutants in *C. elegans* (unpublished results), we have identified an EHEC mutant strain YQ033 (EDL933 *rfaD::Tn5*) in which the *rfaD* gene was disrupted by a Tn5 transposon insertion in the EHEC O157:H7 strain EDL933. In order to confirm the notion that ablation of the *rfaD* gene attenuated the toxicity of EHEC, we generated an EDL933 isogenic mutant with *rfaD* deletion (EDL933:Δ*rfaD*) and tested the toxicity of the *rfaD* deletion mutant along with the Tn5 transposon mutant in *C. elegans*. As shown in Figure 2A, *C. elegans* N2 animals fed with EHEC O157:H7 strain EDL933 had significantly shorter life spans than if fed with *E. coli* strain OP50 (*P* < 0.001), the standard nonpathogenic food source for *C. elegans*. However, when fed with both EDL933:Δ*rfaD* (*P* < 0.001) and EDL933 *rfaD::Tn5* (*P* < 0.001), *C. elegans* N2 animals lived significantly longer than animals fed with wild-type EDL933. These results suggest that disruption of the core LPS biosynthesis genes, including *rfaD*, confer less virulence of EHEC toward *C. elegans*.

In order to strengthen our hypothesis that altering the EHEC core LPS biosynthesis genes attenuated toxicity, we generated the EDL933 isogenic mutants with *rfaE* (also known as *gmhC* and encoding the upstream enzyme of RfaD in the core LPS synthesis pathway, Figure 1B) deletion (EDL933:Δ*rfaE*) or *rfaC* (which encodes the downstream enzyme of RfaD, Figure 1B) deletion (EDL933:Δ*rfaC*), and tested their virulence toward *C. elegans* (Figures 2B,C, respectively). N2 animals lived significantly longer feeding on EDL933:Δ*rfaE* plates (*P* < 0.001) and EDL933:Δ*rfaC* plates (*P* < 0.001) compared to those on the wild-type EDL933 plates. Moreover, the survival curve of N2 animals fed with EDL933:Δ*rfaD* was similar to that with EDL933:Δ*rfaE* or EDL933:Δ*rfaC.* Taken together, the data demonstrated that the EHEC core LPS is required for its toxicity in *C. elegans*.

In order to reconfirm the role of *rfaD* in EHEC virulence against *C. elegans*, we complemented the *rfaD* deletion in the EDL933:Δ*rfaD* mutant by transformation of the RfaD protein expression plasmid, *prfaD*. As a result, the survival curve of N2 animals fed with the *prfaD* complement strain, EDL933:Δ*rfaD*-*prfaD*, was significantly different from that of animals fed with EDL933:Δ*rfaD* (*P* < 0.001), and was similar to that of animals fed with wild-type EDL933 (*P* = 0.128) (Figure 2D). Our data demonstrated that the attenuated toxicity of the *rfaD* deletion mutant in *C. elegans* was completely rescued by the RfaD expression plasmid. An interpretation for the *rfaD* deletion in EDL933 resulting in the attenuation of its virulence is that disruption of RfaD and the core LPS biosynthesis affects the general physiology of EHEC, i.e., the growth of bacterial cells. In order to address this question, we directly analyzed the growth kinetic curves of wild-type EDL933, EDL933:Δ*rfaD* and EDL933:Δ*rfaD*-*prfaD* bacteria. As shown in Figure 2E, the growth kinetics of EDL933:Δ*rfaD* mutant exhibited no significant difference to that of wild-type EDL933 and EDL933:Δ*rfaD*-*prfaD* bacteria. Taken together, our results suggested that ablation of *rfaD* leading to attenuation of EHEC is due to an unidentified mechanism other than retardation of bacterial growth.

To examine whether disruption of the *rfaD* gene in EDL933 affects its LPS composition, we also isolated the LPS from OP50, EDL933, EDL933 *rfaD::Tn5*, EDL933:Δ*rfaD*, and EDL933:Δ*rfaD-prfaD* bacteria. These LPS samples were resolved and visualized (Figure 2F). EDL933 mutant strains with transposon insertion and deletion in the *rfaD* gene lacked the O-antigen, but the wild-type EDL933 and EDL933:Δ*rfaD-prfaD* bacteria contained intact O-antigen. These data reconfirmed that the LPS structures were altered in these EHEC *rfaD* mutants. Moreover, the nonpathogenic *E. coli* OP50 also lacked O-antigen, which was in agreement with a previous report (Darby, 2005). In order to examine the role of *rfaD* gene *per se* in the virulence of *E. coli* and to test whether *C. elegans* lives longer feeding on the *rfaD* mutant of nonpathogenic *E. coli*, we generated the OP50 isogenic mutant with *rfaD* deletion (OP50:Δ*rfaD*) and tested the virulence. N2 animals fed with OP50:Δ*rfaD* exhibited a similar survival curve to those fed with wild-type OP50 (*P* = 0.795) (Figure S1). Taking the above data together, our results indicated that the genes involved in core LPS biosynthesis of EHEC strain EDL933, especially *rfaD*, are required for EDL933 infection in *C. elegans*.

In order to test whether the role of *rfaD* in the virulence against *C. elegans* is general to *E. coli* O157:H7, we also generated *rfaD* mutant in a clinical *E. coli* O157:H7 isolated strain HER1266 (HER1266:Δ*rfaD*). As shown in Figure S2, *C. elegans* fed with HER1266:Δ*rfaD* exhibited a significantly longer life span (*P* < 0.001) compared to that fed with wild-type HER1266. Moreover, the *prfaD* complement strain (HER1266:Δ*rfaD-pfraD*) was significantly more toxic than the HER1266:Δ*rfaD* (*P* < 0.001) and showed no statistical difference compared to the wild-type HER1266 (*P* = 0.088). Collectively, our data corroborated the notion that deletion of the *rfaD* gene attenuates the toxicity of *E. coli* O157:H7 in *C. elegans*. Moreover, the other two LPS biosynthesis mutants, EDL933 *waaI::Tn5* (*waaI* encodes the α-1,3-D-galactosyltransferase and acts downstream of RfaD in O-oligosaccharide biosynthesis) and EDL933 *wbdP::Tn5* (*wbdP* encodes a putative glycosyltransferase in O-antigen biosynthesis) also exhibited significant attenuated toxicity toward *C. elegans* (Figure S3). All together, our results indicated that the genes involved in LPS biosynthesis, including *rfaD*, are required for EHEC infection in *C. elegans*.

### Mutation in *rfaD* confers reduced bacterial colonization and less microvillar actin rearrangement in the intestine of *C. elegans*

**Figure 3:**
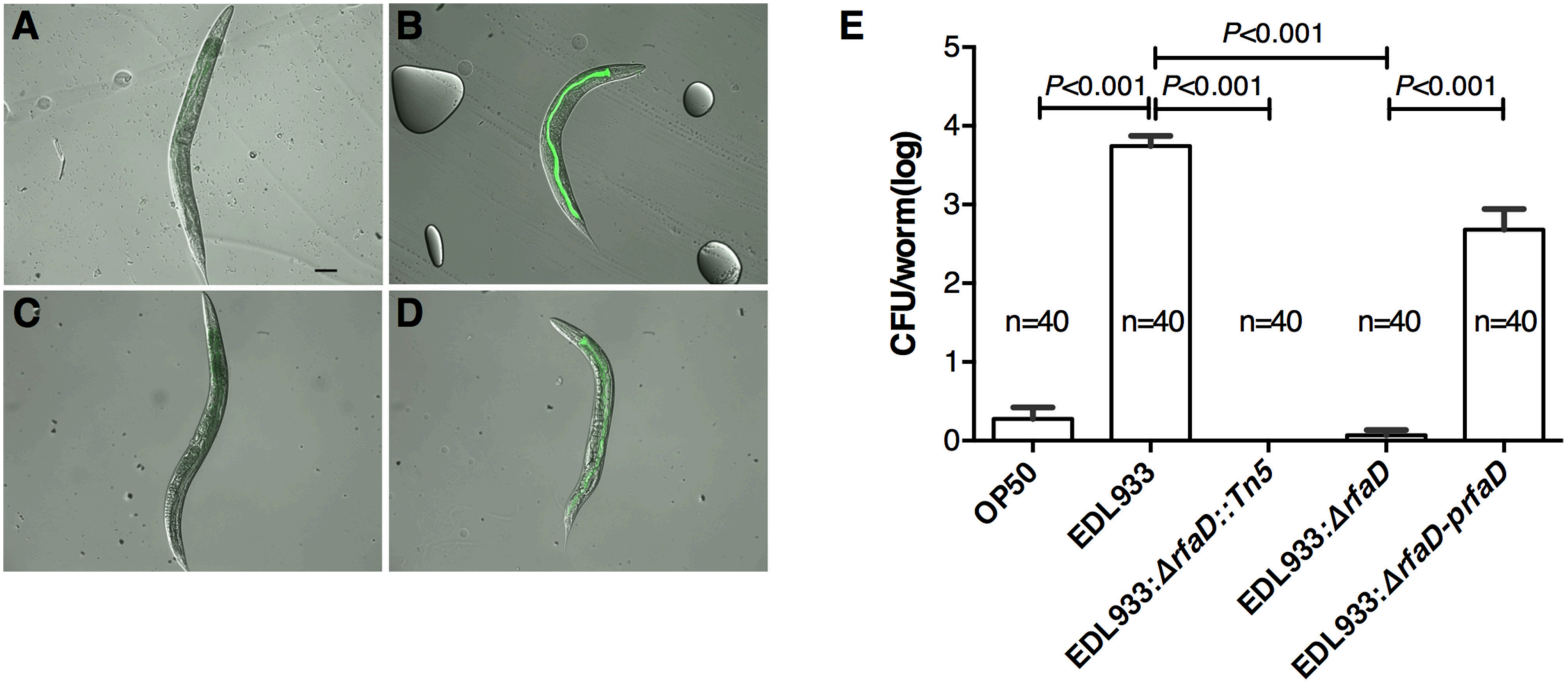
**Mutation in *rfaD* reduces EDL933 colonization in *C. elegans.*** Images of wild-type N2 nematodes fed with GFP-labeled OP50 **(A)**, EDL933 **(B)**, EDL933: Δ*rfaD* **(C)** or EDL933:Δ*rfaD-prfaD* **(D)** for 1 days at 20°C and then chased with non GFP-labeled OP50 for 3 days at 20°C, respectively. Animals previously exposed on GFP-labeled EDL933 and EDL933:Δ*rfaD-prfaD* plates showed significant GFP signals in their intestines. Animals fed with wild-type EDL933 and EDL933:Δ*rfaD-prfaD* exhibited unhealthy apperances with smaller and paler body compared to animals fed with OP50 and EDL933:Δ*rfaD.* Representative images are shown, and the scale bar represents 100 μm. **(E)** The number of bacteria colonized in *C. elegans* was determined by the colony forming units (CFU) assay. Values represent the means of three independent assays, and error bars indicate the standard deviations. *P*-values denote the results of statistical analysis. The total numbers of animals tested in each group are indicated by n.

*E. coli* O157:H7 is an enteric pathogen, which can colonize in the gastrointestinal tract of humans. We have demonstrated that EDL933 colonizes in the intestine of *C. elegans* (Chou et al., 2013). Therefore, we next examined whether EDL933:Δ*rfaD* reduces its colonization in the intestine of *C. elegans*. N2 animals were fed with green fluorescent protein (GFP) labeled OP50, EDL933, EDL933:Δ*rfaD* or EDL933:Δ*rfaD-prfaD* for 1 day and transferred to normal non-GFP-labeled OP50 for another 3 days. As shown in Figures 3A–D, the N2 animals pulsed previously with GFP-labeled EDL933 and GFP-labeled EDL933:Δ*rfaD-prfaD* showed enhanced GFP signals in their intestinal tract after chasing with non-GFP-labeled OP50. However, GFP signals were not detectable in the alimentary tract of the animals fed with either GFP-labeled OP50 or GFP-labeled EDL933:Δ*rfaD* previously. N2 animals fed with wild-type EDL933 and EDL933:Δ*rfaD-prfaD* were smaller, paler and had an unhealthy apperance compared to animals fed with OP50 and EDL933:Δ*rfaD*. Moreover, the average colony formation units (CFU) of the animals pulsed with GFP-labeled EDL933:Δ*rfaD* or EDL933 *rfaD::Tn5* for 1 day and then chased with non-GFP-labeled OP50 for 3 days were significantly reduced (*P* < 0.001) compared to that of the control GFP-labeled EDL933 fed animals (Figure 3E). The *rfaD* complementation group showed a significantly increased intestinal colonization phenotype compared to the *rfaD* mutants (EDL933:Δ*rfaD* and EDL933 *rfaD::Tn5*). These data demonstrated that mutations in *rfaD* reduce the intestinal colonization ability of EDL933 in *C. elegans*.

**Figure 4:**
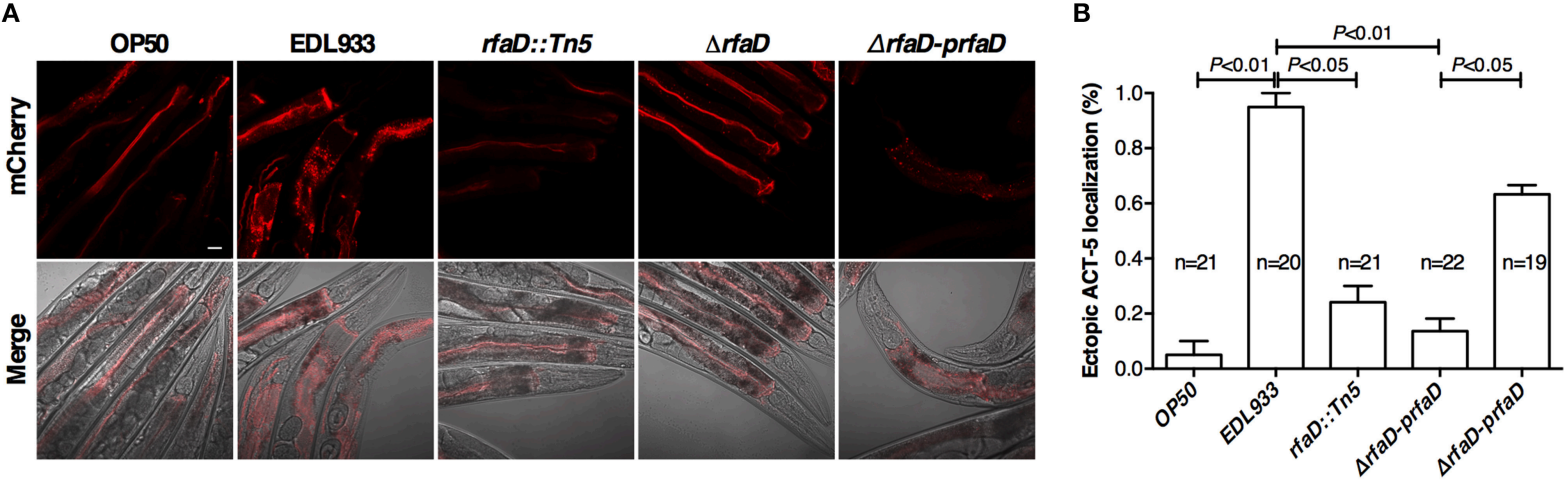
**Mutations in *rfaD* reduce EHEC-induced intestinal microvillar actin rearrangement in *C. elegans*. (A)** The confocal images showed the mCherry-tagged ACT-5 (intestinal microvillar actin) of GK454 transgenic animals fed with OP50, EDL933, EDL933 *rfaD::Tn5* (*rfaD::Tn5*), EDL933:Δ*rfaD* (Δ*rfaD*) or EDL933:Δ*rfaD-prfaD* (Δ*rfaD-prfaD*) for 4 days respectively. Ectopic localization of the mCherry::ACT-5 signals from the apical membrane to the cytoplasm of the intestinal cells were significantly increased in the EDL933 treated group. Upper panels show mCherry images; lower panels are the merge images of the DIC and mCherry signals. Representative images are shown, and scale bar represents 10 μm. **(B)** Quantifications of animals with the EHEC-induced ectopic mCherry::ACT-5 signal were determined. Values represent the means of three independent assays, and error bars indicate the standard deviations. *P*-values denote the results of statistical analysis.

EHEC induced host A/E (attaching and effacing) lesion in intestinal tract, which caused cytoskeleton actin rearrangement in host intestinal cells. Our previous report demonstrates that the transgenic *C. elegans* animals expressing mCherry-tagged ACT-5 can serve as a good model to monitor EHEC-induced microvillar actin rearrangement in intestinal cells (Chou et al., 2013). We examined the mCherry signals of the transgenic *C. elegans* animals fed with wild-type EDL933 and *rfaD* mutants by confocal microscopy. As shown in Figure 4A, the mCherry::ACT-5 animals fed with wild-type EDL933 for 4 days at 20°C exhibited significant ectopic mCherry::ACT-5 signals from apical plasma membrane to the cytoplasm. However, the mislocalozation of ACT-5 significantly decreased in the animals fed with the *rfaD* mutants (EDL933:Δ*rfaD* and EDL933 *rfaD::Tn5*) compared to that with wild-type EDL933. The transgenic animals fed with EDL933:Δ*rfaD-prfaD* showed a similar phenotype to that of wild-type EDL933. Quantifications of animals with the ectopic mCherry::ACT-5 signal after the exposure to wild-type EDL933 and *rfaD* mutants were also determined (Figure 4B). The data showed that the *rfaD* mutants are significantly less virulent to *C. elegans* animals in terms of the induction of A/E lesions. Taking the above data together, we demonstrated that the *rfaD* mutations confer attenuation of EHEC to *C. elegans in vivo*.

### Disruption of *rfaD* reduces bacterial colonization in the intestine of mouse

**Figure 5:**
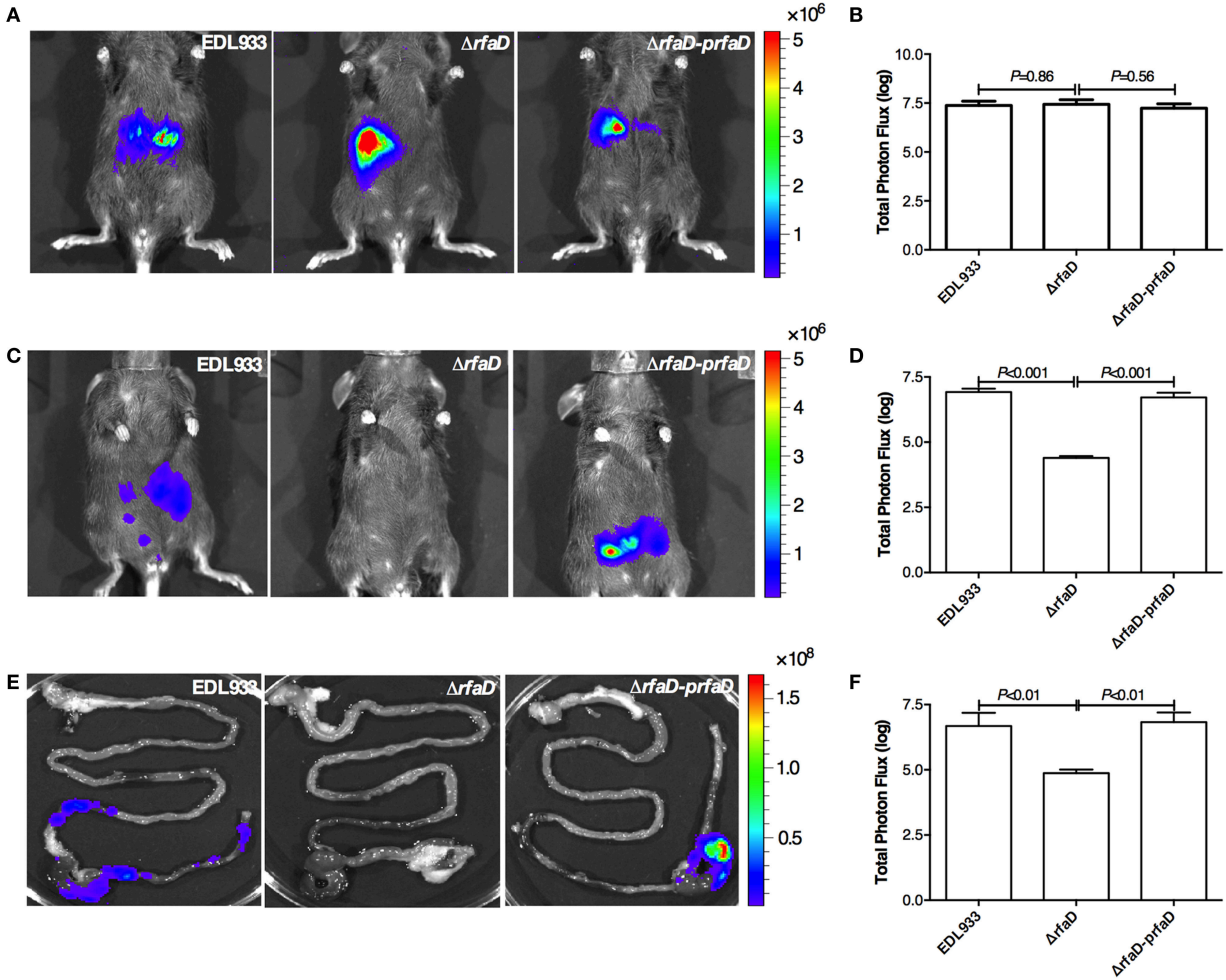
**Disruption of *rfaD* reduces EDL933 colonization in the intestines of mice. (A)** Represent images of mice inoculated with bioluminescence-labeled wild-type EHEC EDL933 (EDL933), EDL933:Δ*rfaD* (Δ*rfaD*), and EDL933:Δ*rfaD-prfaD* (Δ*rfaD-prfaD*) 1 h post infection. **(B)** Quantification of bioluminescence intensity of mice infected with EDL933, EDL933:Δ*rfaD* and EDL933:Δ*rfaD-prfaD* 1 h post infection. **(C)** Represent images of mice inoculated with bioluminescence-labeled wild-type EHEC EDL933 (EDL933), EDL933:Δ*rfaD* (Δ*rfaD*), and EDL933:Δ*rfaD-prfaD* (Δ*rfaD-prfaD*) 2 days post infection. **(D)** Quantification of bioluminescence intensity of mice infected with EDL933, EDL933:Δ*rfaD* and EDL933:Δ*rfaD-prfaD* 2 days post infection. **(E)** Represent images of intestinal tissues of mice infected with bioluminescence-labeled wild-type EHEC EDL933 (EDL933), EDL933:Δ*rfaD* (Δ*rfaD*), and EDL933:Δ*rfaD-prfaD* (Δ*rfaD-prfaD*). **(F)** Quantification of bioluminescence signals of intestinal tissues of bioluminescent EHEC infected mice 2 days post infection. The color scale represents the radiance (p/sec/cm^2^/sr). Representative images are shown. All experiments were conducted independently three times with 3 animals each time, and error bars indicate the standard deviations.

We next tested whether deletion of the *rfaD* gene in EHEC also confers less intestinal colonization in mammals. To this end, we generated the bioluminescence-labeled wild-type EDL933, EDL933:Δ*rfaD* and EDL933:Δ*rfaD-prfaD* bacteria by transformation of the luciferase expressing plasmid, pWF279, and examined the bioluminescent signal by the non-invasive *in vivo* imaging system (IVIS) after oral gavage of approximately 10^9^ CFU bioluminescent bacteria into 6 week-old female C57BL/6 mice pretreated with streptomycin (Figure 5). The mice infected intragastrically with bioluminescence-labeled wild-type EDL933, EDL933:Δ*rfaD* and EDL933:Δ*rfaD-prfaD* exhibited similar bioluminescent signal levels 1 h post infection (Figures 5A,B). While, the mice infected with bioluminescence-labeled wild-type EDL933 and EDL933:Δ*rfaD-prfaD* revealed significant bioluminescent signals 2 days post infection However, the bioluminescent signals of mice infected with EDL933:Δ*rfaD* were significantly decreased after 2 days post infection (Figures 5C,D).

Next, we determined the localization of these bioluminescence-labeled bacteria. The intestines of mice 2 days post infection were removed and imaged by IVIS *ex vivo.* As shown in Figure 5E, the bioluminescent signals of wild-type EDL933 and EDL933:Δ*rfaD-prfaD* bacteria were detected in the cecum and colon, which suggest that EHEC can colonize in the cecum and colon of mice for at least 2 days, while the bioluminescent signal was not detectable in the intestines of mice infected by EDL933:Δ*rfaD*. The quantification of bioluminescent signals showed similar results, i.e., the bioluminescent signals were significantly decreased in the EDL933:Δ*rfaD* treated mice compared to the wild-type EDL933 (*P* < 0.01) and EDL933:Δ*rfaD-prfaD* (*P* < 0.01) treated groups (Figure 5F). Taken together, these data demonstrated that the abolishment of functional *rfaD* reduces EDL933 colonization to the intestine, specifically in the cecum and colon, of mouse. Given that EHEC is considered less virulent to mouse when infected orally (Mohawk and O'Brien, 2011), our data also suggested that loss of core LPS in EHEC may increase its susceptibility to host intestinal immunity and therefore accelerate the elimination rate of bacterial cells from the host body.

### Deletion of *rfaD* increases the susceptibility of EHEC to human serum killing

**Figure 6:**
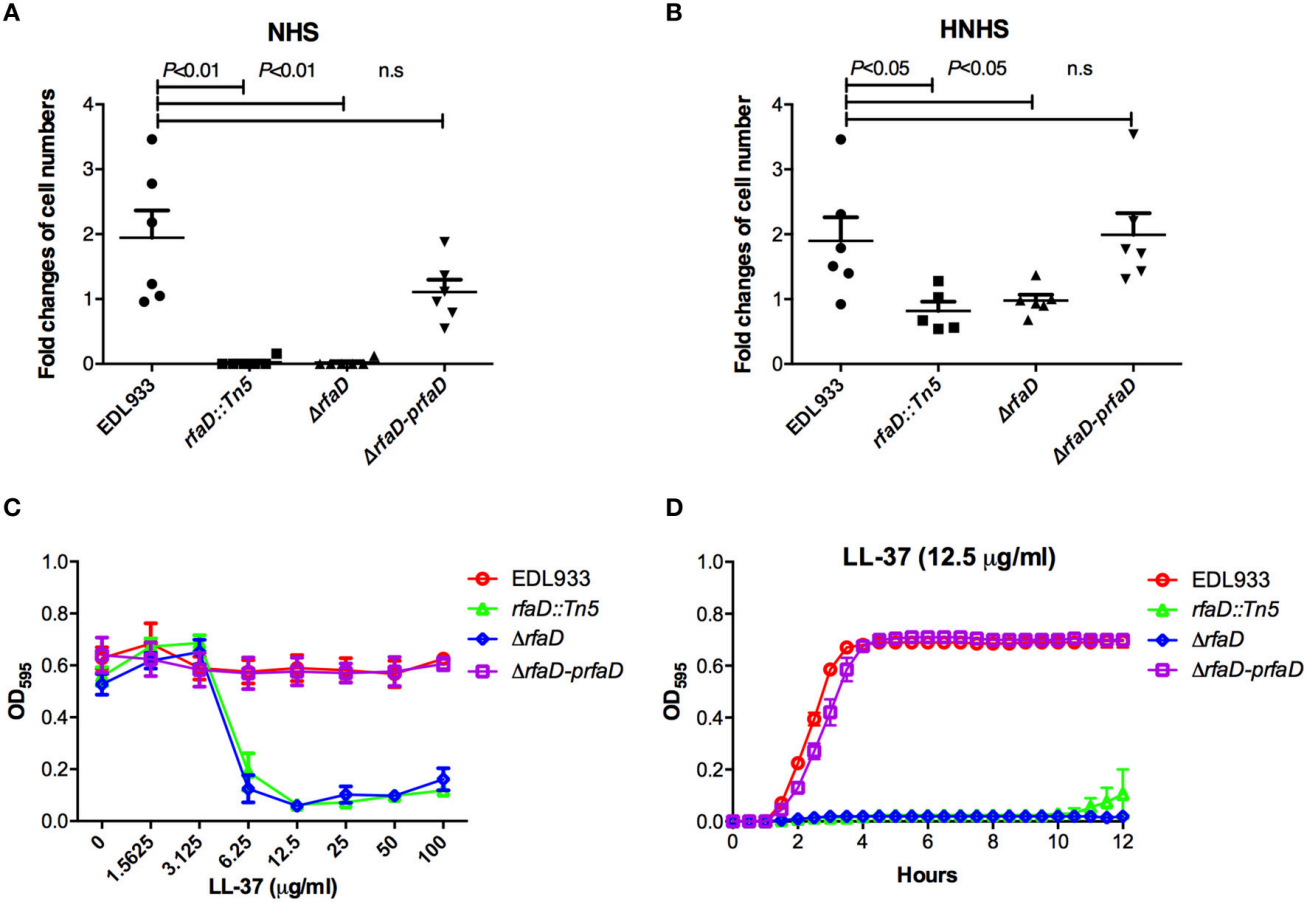
**Deletion of EHEC *rfaD* increases its susceptibility to human serum killing and the antimicrobial peptide LL-37**. The fold changes of bacterial cell numbers of wild-type EDL933, EDL933 *rfaD::Tn5*, EDL933:Δ*rfaD* and EDL933:Δ*rfaD-prfaD* after incubation in 10% normal human serum (NHS) **(A)** or 10% heat-inactivated normal human serum (HNHS) **(B)** at 37°C for 60 min were measured. Experiments were conducted independently at least three times, and error bars indicate the standard deviations. **(A)** The *rfaD* transposon mutant (*rfaD::Tn5, P* = 0.001) and *rfaD* deletion mutant (Δ*rfaD, P* = 0.001) showed significant decreased survival ratio compared to that of wild-type EDL933 (EDL933) in 10% NHS. The *rfaD* complement bacteria (Δ*rfaD-prfaD*) showed comparable survival ratio compared to that of EDL933 (*P* = 0.101) in 10% NHS. **(B)** The *rfaD* transposon mutant (*rfaD::Tn5, P* = 0.031) and *rfaD* deletion mutant (Δ*rfaD, P* = 0.034) showed significantly decreased survival ratio compared to that of wild-type EDL933 (EDL933) in 10% HNHS. The *rfaD* complement bacteria (Δ*rfaD-prfaD*) showed comparable survival ratio compared to that of EDL933 (*P* = 0.854) in 10% HNHS. n.s. indicates no statistical significance. **(C)** The OD_595_ values of wild-type EDL933 (EDL933), EDL933 *rfaD::Tn5* (*rfaD::Tn5*), EDL933:Δ*rfaD* (Δ*rfaD*), and EDL933:Δ*rfaD-prfaD* (Δ*rfaD-prfaD*) cultured with different dose of LL-37 at 37°C for 16 h were monitored. **(D)** The growth curves of EDL933, *rfaD::Tn5*, Δ*rfaD* and Δ*rfaD-prfaD* in the presence of 12.5 μg/ml LL-37 at 37°C.

One characteristic of EHEC virulence is its serum resistance. Several virulence factors encoded by EHEC, including the *iss, traT*, and *stcE* genes, which confer EHEC serum resistance have been documented (Binns et al., 1979, 1982; Lathem et al., 2004). Chain length of LPS has also been reported to affect serum resistance in *Salmonella* (Bravo et al., 2008). However, the correlation between *E coli* O157:H7 LPS and serum resistance remains poorly understood. To assess whether the LPS protects EHEC from serum killing, we monitored the survival rate of EHEC cells in the presence of human serum. The relative survival ratios of EDL933, EDL933 *rfaD::Tn5*, EDL933:Δ*rfaD*, and EDL933:Δ*rfaD-prfaD* in either 10% normal human serum (NHS) or 10% heat-inactivated normal human serum (HNHS) were measured. In HNHS, the complement system in normal serum was inactivated after the pre-incubation of NHS at 56°C for 30 min. As shown in Figure 6A, the wild-type EDL933 cells exhibited a significantly greater survival ratio compared to the *rfaD* transposon mutant (EDL933 *rfaD::Tn5, P* = 0.001) and *rfaD* deletion mutant (EDL933:Δ*rfaD, P* = 0.001) in 10% NHS at 37°C for 1 h. The wild-type EDL933 cells still replicated (1.94 fold increase) in 10% NHS, but the EDL933 *rfaD::Tn5* and EDL933:Δ*rfaD* cells were killed in the presence of NHS (0.03 fold and 0.02 fold, respectively). The *rfaD* complement (EDL933:Δ*rfaD-prfaD*) cells rescued the serum resistance defect of the *rfaD* mutation and showed similar survival ratio (1.21 fold increase, *P* = 0.101) compared to wild-type EDL933 cells. Together, our data demonstrated that *rfaD* is required for EHEC serum resistance.

When these bacterial cells were cultured in 10% HNHS (Figure 6B), the wild-type EDL933 (1.90 fold increase) and EDL933:Δ*rfaD-prfaD* cells (1.99 fold increase) still replicated normally. The survival ratios of these two strains in 10% HNHS and 10% NHS were similar (EDL933, *P* = 0.937; EDL933:Δ*rfaD-prfaD, P* = 0.05), which supports the notion that EHEC is serum resistant. Moreover, both the *rfaD* transposon mutant and deletion mutant showed significantly greater ability to survive in 10% HNHS (0.82 fold and 0.94 fold, respectively) than in 10% NHS (0.03 fold and 0.02 fold, respectively). These data demonstrated that *rfaD* is required for the defense of the complement system in human serum. Interestingly, the survival ratios of the *rfaD* mutants (EDL933 *rfaD::Tn5, P* = 0.031 and EDL933:Δ*rfaD, P* = 0.034) were still significantly decreased compared to the wild-type EDL933 in 10% HNHS (Figure 6B). These data suggested that the *rfaD* mutants are still hypersusceptible to the complement-independent killing effect of normal human serum compared to wild-type EDL933.

### Disruptions of *rfaD* increase the susceptibility of EHEC to AMPs *in vitro*

Antimicrobial peptides (AMPs) are short peptides that inhibit bacterial cell growth and constitute a host innate immune system against bacterial infection. AMPs target the bacterial membrane and disrupt the membrane integrity of bacteria cells, and the lack of the protection of LPS barrier leads AMPs to target the bacteria membrane more easily (Rosenfeld and Shai, 2006; Bahar and Ren, 2013). It has been reported that mammalian serum contains a variety of AMPs, including Cathelicidin, and still maintains antibiotic activity after 65°C treatment (Mahoney et al., 1995), which resembles the complement-independent innate immunity that inhibited the growth of *rfaD* mutants in the 56°C heat-inactivated human serum (Figure 6B). In order to test whether disruption of *rfaD* could alter the susceptibility of EHEC to AMPs, we cultured the wild-type EDL933 and *rfaD* mutants in the presence of LL-37, a short peptide with the bactericidal function derived from the Cathelicidin family of AMP (Vandamme et al., 2012; Wang, 2014), and examined their growth kinetics *in vitro*. The minimum inhibitory concentrations (MIC) of LL-37 against the *rfaD* mutants, including EDL933 *rfaD::Tn5* (6.25 μg/ml), and EDL933:Δ*rfaD* (6.25 μg/ml), were significantly lower than that of wild-type EDL933 (>100 μg/ml) and EDL933:Δ*rfaD-prfaD* (>100 μg/ml) (Figure 6C). The growth kinetics of bacteria cultured with LL-37 also showed that LL-37 significantly inhibits the replication of EDL933 *rfaD::Tn5*, and EDL933:Δ*rfaD* cells (Figure 6D). Moreover, the *rfaD* mutations also increased the susceptibility of EDL933 to the other two AMPs, Polymyxin B and Colistin (all *P* < 0.05, Table S3). Together, our data demonstrated that disruption of *rfaD* increases the susceptibility of EHEC to AMPs *in vitro*.

### Deletion of *rfaD* confers the hypersusceptibility of EHEC to host intestinal immunity *in vivo*

**Figure 7:**
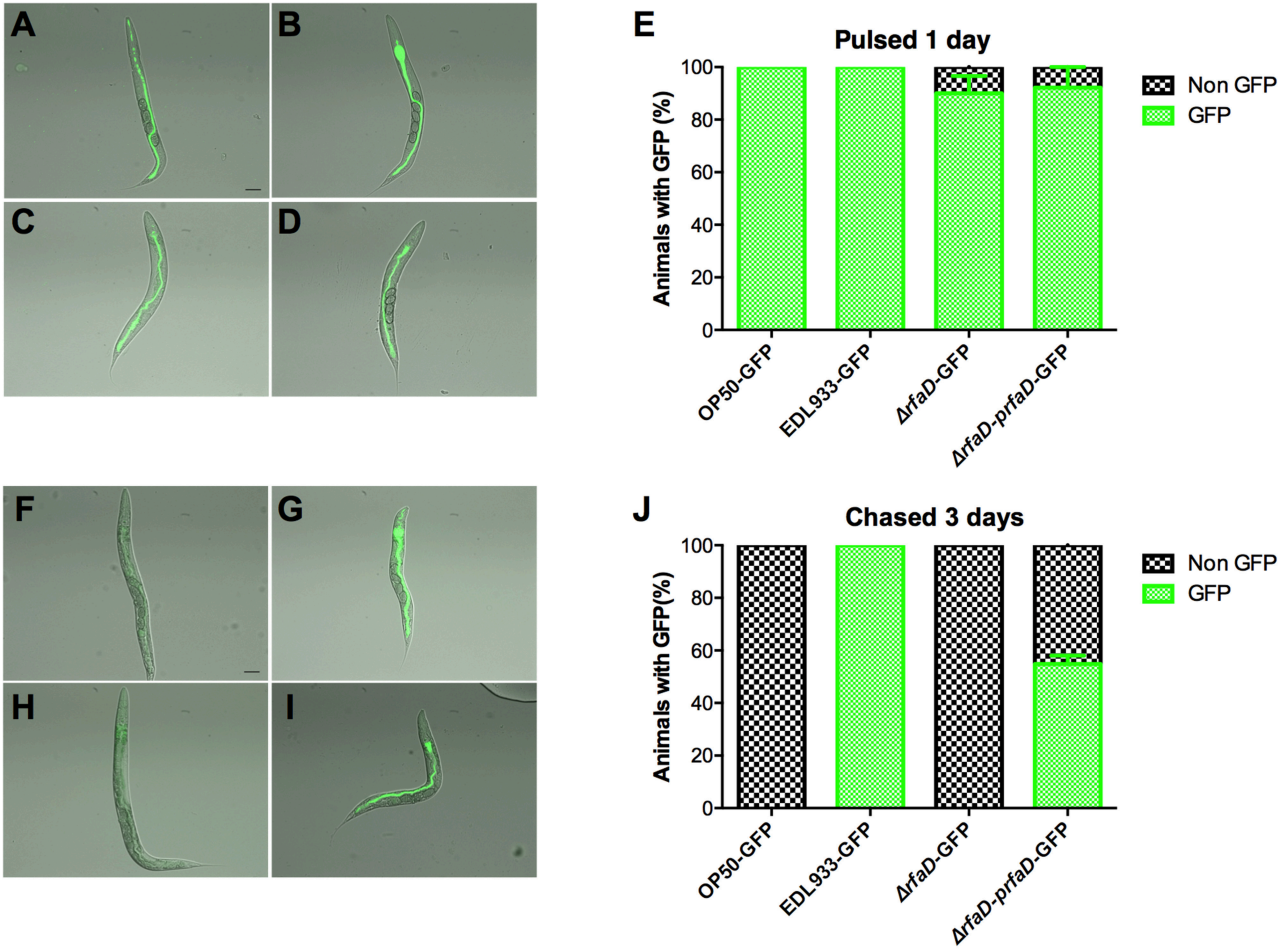
**The *rfaD* mutation increases the susceptibility of EHEC to host intestinal innate immunity**. Images of DA597 pharynx defect *C. elegans* fed with **(A)** GFP-labeled OP50 (OP50-GFP), **(B)** EDL933 (EDL933-GFP), **(C)** EDL933:Δ*rfaD* (Δ*rfaD*-GFP), or **(D)** EDL933:Δ*rfaD-prfaD* (Δ*rfaD-prfaD*-GFP) at 20°C for 1 day. **(E)** Percentage of GFP-labeled bacteria **(A–D)** fed DA597 animals with GFP signals in intestine was calculated. Images of DA597 animals fed with **(F)** GFP-labeled OP50, **(G)** EDL933, **(H)** EDL933:Δ*rfaD*, or **(I)** EDL933:Δ*rfaD-prfaD* at 20°C for 1 days and chased on normal non-GFP OP50 plates for 3 days. **(J)** Percentage of GFP-labeled bacteria **(F–I)** fed DA597 animals with GFP signals in intestine was calculated. All experiments were conducted independently at least three times, and error bars indicate the standard deviations. The scale bar represents 100 μm.

We showed that the GFP-labeled wild-type EDL933 can replicate and colonize in the intestinal lumen of *C. elegans*; however, the GFP-labeled *rfaD* mutants cannot (Figure 3). Given that *C. elegans* is a bacterivore and most of the bacteria cells ingested are ground up by the pharynx of *C. elegans*, another interpretation of these results is that the physiological integrity of the outer membrane in the *rfaD* mutants is weak and therefore makes them more vulnerable to the grinding force of the pharynx, other than simply increasing their susceptibility to innate immunity in the intestine of *C. elegans*. In order to test whether *rfaD* mutants are directly hypersusceptible to the intestinal antimicrobial immunity *in vivo*, we monitored the replication and colonization of the *rfaD* mutants in the pharynx-defect *C. elegans* strain DA597 (Figure 7). The DA597 animals contain two mutations in the *phm-2(ad597)* allele that result in abnormal position and function of the pharyngeal grinder (Avery, 1993). When DA597 animals were pulsed with GFP-labeled OP50, wild-type EDL933, EDL933:Δ*rfaD*, or EDL933:Δ*rfaD-prfaD* bacteria for 1 day, most of the animals exhibited GFP signals in their intestine and there was no significant difference among different bacteria-treated groups (Figures 7A–E). The data suggested that these GFP-labeled bacteria can pass into the intestinal lumen without being damaged by the pharynx in DA597 animals. However, when these GFP-labeled bacteria infected animals were further chased with non-GFP-labeled OP50 for another 3 days (Figures 7F–J), only DA597 animals infected previously by GFP-labeled wild-type EDL933 and EDL933:Δ*rfaD-prfaD* still maintained significant GFP signals in their intestines. However, the GFP signals were totally abolished in the GFP-labeled OP50 and EDL933:Δ*rfaD*-infected groups. These data support the notion that the EDL933:Δ*rfaD*, similar to the non-pathogenic OP50, is hypersensitive to the intestinal innate immunity in *C. elegans*, and are therefore quickly killed and eliminated by the animals *in vivo*. This phenomenon is similar to that in mice described in Figure 5. Together, our results suggested that disruptions of the *rfaD* gene increased the susceptibility of EHEC to host intestinal immunity.

## Discussion

Our genetic analyses in the present study demonstrate that *rfaD* and genes involved in the LPS biosynthesis are required for EHEC infection in *C. elegans.* Disruption of *E. coli* O157:H7 LPS attenuates its toxicity to *C. elegans.* The *E. coli* O157:H7 *rfaD* mutants are significantly less virulent to *C. elegans* animals, as shown by significantly reduced bacterial colonization and microvillar actin rearrangement in the alimentary tract of *C. elegans*. Moreover, mutation of *rfaD* diminishes the colonization of *E. coli* O157:H7 in the intestine, specifically in the cecum and colon, in mouse. Our mechanistic study demonstrates that the attenuated phenotype of *E. coli* O157:H7 *rfaD* mutant to animal hosts may, at least in part, be due to an increase in susceptibility to antimicrobial peptides and host intestinal immunity *in vivo*.

The enzymatic product of RfaD, ADP-L-*glycero*-D-*manno*-heptose, links core LPS and O-antigen to Kdo_2_-lipid A on the EHEC outer membrane (Figure 1). It has been reported that inhibition of the KdtA (also called WaaA), which links the ADP-L-*glycero*-D-*manno*-heptose to Kdo_2_-lipid A for the core-lipid A formation, causes the accumulation of Lipid A Kdo_2_ disaccharide on the outer membrane of *E. coli* (Belunis et al., 1995). Given that the RfaD is the last step enzyme for ADP-L-*glycero*-D-*manno*-heptose and inner core LPS biosynthesis, inactivation of RfaD may also result in the accumulation of Kdo_2_-lipid A on the EHEC outer membrane. Of note, Kdo_2_-lipid A has been reported to stimulate the innate immune system through the Toll-like receptor 4 (TLR4) signaling pathway (Raetz et al., 2006; Sims et al., 2010; Kim et al., 2015), and it has been suggested the *E. coli rfaD* mutants that produce more Kdo2-lipid A could be a good base strain for developing bacterial vaccine adjuvant (Wang et al., 2014). Our results demonstrate that the EHEC *rfaD* mutants are significantly attenuated to animal hosts, including *C. elegans* and mouse, *in vivo*, therefore testing whether the EDL933:Δ*rfaD* mutants can activate the innate immune response is warranted and the potential for these mutants in EHEC vaccine development is suggested.

In metazoans, including *C. elegans* and mammals, host antimicrobial peptides (AMPs) are crucial components of the innate immune system (Irazoqui et al., 2010; Nakatsuji and Gallo, 2012; Pukkila-Worley and Ausubel, 2012). Humans have approximately one hundred AMPs, and these AMPs have been found on the mucus surface layer of a variety of tissues, including intestine (Wang, 2014). It has been reported that the AMP Cathelicidin mediates innate intestinal defense against colonization with epithelial adherent bacterial pathogens, including *Escherichia coli* O157:H7 (Iimura et al., 2005), and protects mice from EHEC-mediated disease (Chromek et al., 2012). Our *in vitro* and *in vivo* data in Figures 5–7, which suggest that the EHEC *rfaD* mutants are hypersensitive to AMPs and confer less intestinal colonization, are in agreement with these reports. However, it also has been reported that the EHEC outer-membrane protease OmpT can degrade the human AMP LL-37 and has evolved as a strategy through which EHEC can resist AMPs (Thomassin et al., 2012). Our data demonstrated that targeting RfaD and core LPS biosynthesis can further sensitize the EHEC cells to AMP killing and host innate immunity, which are also in agreement with the recent findings (Ho and Waldor, 2007; Sheng et al., 2008; Miyashita et al., 2012; Youn et al., 2013). Therefore, future testing of whether inhibiting RfaD activity would affect the expression of OmpT on the outer-membrane of EHEC is also clinically relevant.

Bacterial pathogens that are resistant to antibiotics have become an urgent worldwide issue, and the antibiotic resistance of EHEC has also been reported (Venturini et al., 2010; Goldwater and Bettelheim, 2012). Pathogens resistant to currently available antibiotics are increasing drastically, not to mention that the treatment of EHEC infection by canonical antibiotics is contraindicated. For decades, targeting LPS biosynthesis and/or export has been considered as an attractive strategy for the treatment of Gram-negative pathogens infection (Cipolla et al., 2011; Walsh and Wencewicz, 2014). However, antibacterial drugs with this mechanism of action are still under development and have not yet been used clinically. It has not escaped our notice that the EHEC *rfaD* deletion strains are deep rough LPS mutants (Figure 2F), therefore another interpretation of our results for the attenuated toxicity of these EHEC LPS mutants *in vivo* could attribute to their general weakness. Nevertheless, the replication rate/growth kinetics of the *rfaD* mutant remained unaffected *in vitro* (Figure 2E). These data suggest that the inhibition of RfaD, in part, can sensitize EHEC to host intestinal immunity and/or microenvironment and enhance their clearance by host *in vivo*. Here, our study highlights the potential of targeting RfaD or the other enzymes in the LPS biosynthesis pathway, if druggable, as alternative therapeutic measure by which to combat EHEC infection.

## Author contributions

CK, JC, HC, CT, TH, WS, SW, TC, and CC conceived and designed the experiments. CK, TH, SW, and TC performed the experiments. CK, JC, HC, CT, SW, TC, and CC analyzed the data. JC, HC, CT, PL, and WS contributed reagents/materials/analysis tools. CK and CC wrote the paper.

## Funding

This work is supported by the Minister of Science and Technology (MOST) grants (101-2311-B-006-005, 102-2311-B-006-005, 102-2321-B-006-022, and 104-2321-B-006-019) to CC.

### Conflict of interest statement

The authors declare that the research was conducted in the absence of any commercial or financial relationships that could be construed as a potential conflict of interest.
