# Supplementary material for: Mutation of the Enterohemorrhagic Escherichia coli Core LPS Biosynthesis Enzyme RfaD Confers Hypersusceptibility to Host Intestinal Innate Immunity In vivo
Source: Front Cell Infect Microbiol. 2016 Aug 12;6:82. doi: 10.3389/fcimb.2016.00082 (PMC4982379; doi:10.3389/fcimb.2016.00082)
Supplement: Supplementary file 1 [file DataSheet1.PDF]

Supplementary information

Materials and methods

Construction of the plasmids

The ADP-L-*glycero*-D-*manno*-heptose-6-epimerase (RfaD) expressing plasmid, pWF208, was constructed by PCR amplification of the 1,230 bp *rfaD* coding region from *E. coli* O157:H7 EDL933 genomic DNA by the forward primer 5'ACATGCATGCTAATATAAGAAAAGAATTATATCATAAATATTAAC 3' and the reverse primer 5'GCGACGCATAAGAGCTCTGCGTCGACGCGT3'. The PCR products were digested with *SphI* and *SalI* and cloned into the 3,423 bp fragment of the plasmid, pQE30 (Qiagen). The luciferase expressing plasmid, pWF279, was modified from pAKlux2 (Karsi and Lawrence, 2007). The anti-kanamycin cassette, *nptII* gene was amplified from pBSL180 by the forward primer 5'CCTATGCATAATAATTCCGCTAGCTTCACG 3' and the reverse primer 5' GCTCCACCGATAATATTCCTGAGTCATACT 3' and the PCR products were ligated to a TA cloning vector (Yeastern Biotech). The *luxCDABE* operon of the pAKlux2 was cloned into the pBBR1MCS4. The *nptII* gene fragment from the TA vector was excised by *NsiI* and *SmaI* digestion and cloned to the *NsiI* and *ScaI* digested pBBR1MCS4 to create the pWF278. The luciferase fragment was then excised from another pAKlux2 by digesting *SpeI-HF* and *ScaI* and cloned to *SpeI-HF* and *SmaI-HF* digested plasmid pWF278 to generate pWF279. For the pWF264, a constitutive GFP expression plasmid with kanamycin resistance was modified from pFVP25.1. The *nptII* gene was amplified from pBSL180 by forward primer, 5'CCTCGATCGAATAATTCCGCTAGCTTCACG 3' and the reverse primer, 5'GCTCCACCGATAATATTCCTGTGCGCAATT3' and the PCR product was ligated to the TA vector. The *nptII* gene fragment from TA vector was digested by *PvuI* and *SmaI* and cloned to *PvuI* and *FspI* digested pFVP25.1.

Construction of the enterohemorrhagic *E. coli* O157:H7 mutants

The EDL933 deletion mutants were made by using the slightly adaptive one-step PCR-based gene inactivation protocol as described (Datsenko and Wanner, 2000). In brief, the homologous upstream and downstream regions of target gene were amplified from the EDL933 genomic DNA by PCR. The kanamycin resistance gene was amplified from pKD4. Each primer sequence contained target-homologous sequences as well as sequences for amplification of the kanamycin gene. These PCR products were electroporated (2500V, 4ms) into *E. coli* O157:H7 EDL933 transformed with the lambda red recombinase plasmid, pKD46 that was previously induced with 10 mM L-arabinose for 5 h. The bacteria were incubated in LB medium for 1 h and then plated on selective medium (LB supplemented with 50 μg/ml of kanamycin) at 37°C.

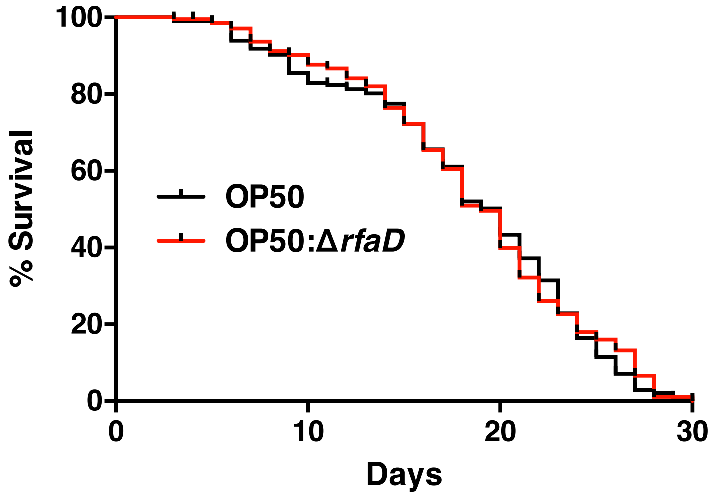

Figure S1. Survival curves of N2 animals fed with *E. coli* OP50 and OP50:*ΔrfaD*.
The survival curve of N2 animals fed with OP50:*ΔrfaD* showed no statistical difference to that of wild-type OP50 (P=0.795). The survival experiment was conducted independently at least three times.

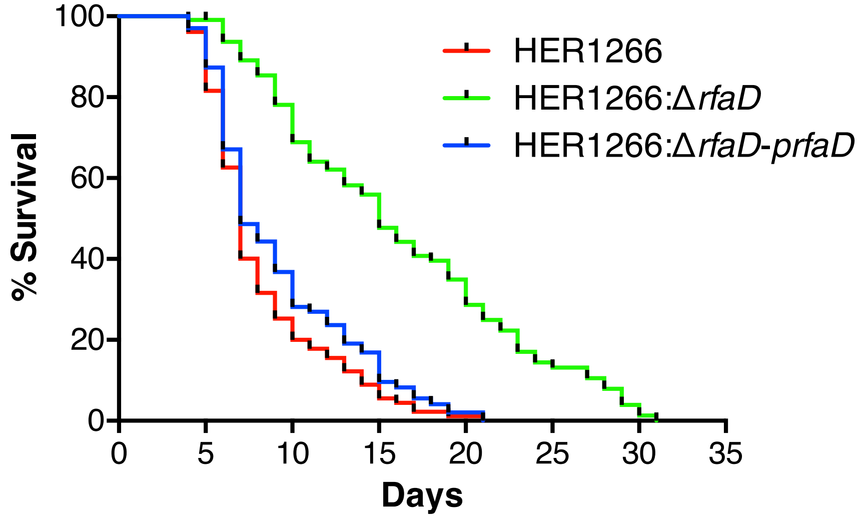

Figure S2. Deletion of *rfaD* attenuates the toxicity of *E. coli* O157:H7 strain HER1266 in *C. elegans.*

N2 animals fed with the EHEC strain HER1266 with *rfaD* isogenic deletion (HER1266:*ΔrfaD*) lived significantly longer than those fed with wild-type HER1266 (HER1266) (P<0.001). The survival curve of *C. elegans* feeding on the *rfaD* complement strain (HER1266:*ΔrfaD-prfaD*) is similar to those fed on the wild-type HER1266 plates (P=0.088). All survival experiments were conducted independently at least three times.

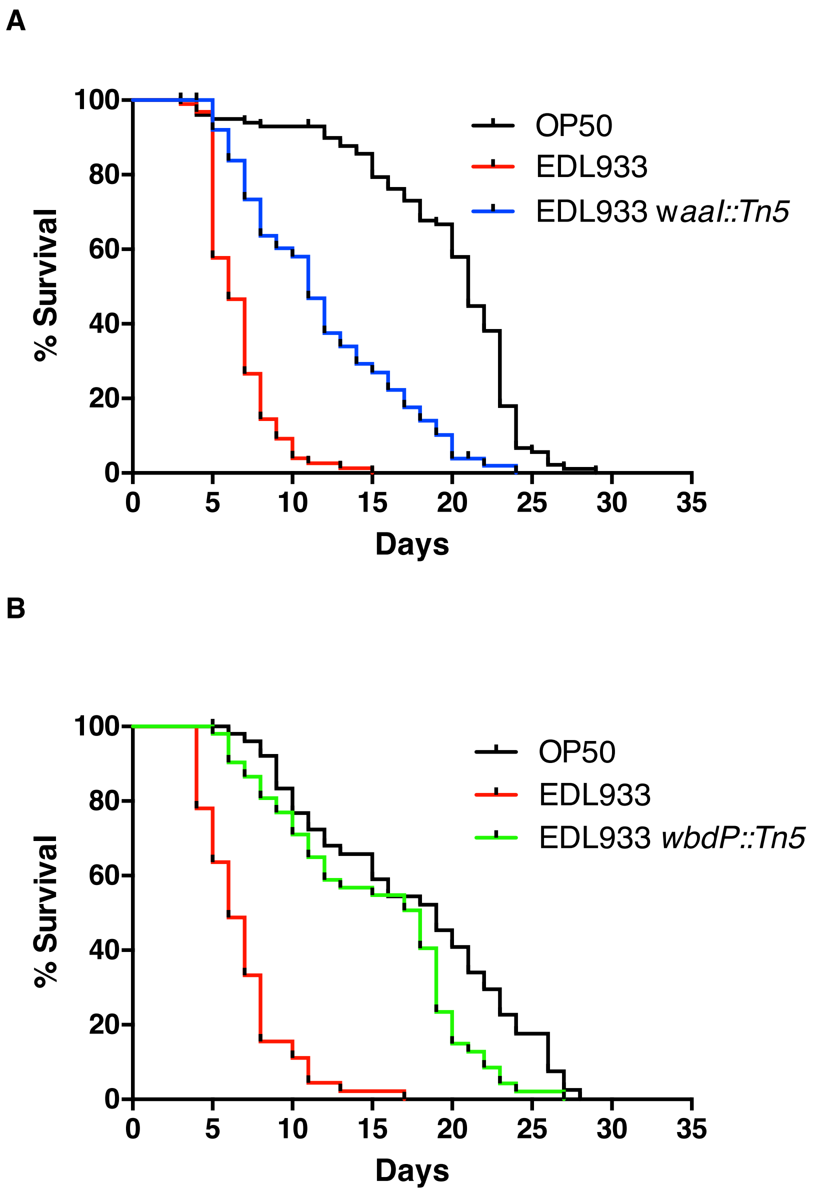

Figure S3. Disruptions of *waaI* and *wbdP* confer attenuated toxicity of EDL933 in *C. elegans.*

(**A-B**) Survival curves of N2 animals fed with *E. coli* strain OP50 (OP50), EHEC strain EDL933 (EDL933), EDL933 with Tn5 transposon insertion in *waaI* (EDL933 *waaI::Tn5*), and EDL933 with Tn5 transposon insertion in *wbdP* (EDL933 *wbdP::Tn5*) were examined. Animals feeding on (A) the EDL933 *waaI::Tn5* mutant plates (P<0.001) and (B) the EDL933 *wbdP::Tn5* plates (P<0.001) lived significantly longer than animals feeding on the wild-type EDL933 plates.

Table S1. Bacteria strains used in this study

Strain	Description	Reference
OP50	*E. coli*. Uracil auxotrophy, standard *C. elegans* laboratory food source.	(Brenner, 1974)
EDL933	*E. coli* O157:H7 isolated from raw hamburger meat implicated in hemorrhagic colitis outbreak.	(Strockbine et al., 1986)
YQ033	EDL933 *rfaD::Tn5*, *rfaD* transposon mutant	This Study
YQ391	EDL933 *waaI::Tn5*, *waaI* transposon mutant	This Study
YQ392	EDL933 *wbdP::Tn5*, *wbdP* transposon mutant	This Study
YQ217	EDL933:*ΔrfaD*, EDL933 isogenic mutant with *rfaD* deletion	This Study
YQ282	EDL933:*ΔrfaE*, EDL933 isogenic mutant with *rfaE* deletion, Kan^r (Kanamycin resistant)	This Study
YQ283	EDL933:*ΔrfaC*, EDL933 isogenic mutant with *rfaC* deletion, Kan^r	This Study
YQ222	EDL933:Δ*rfaD-prfaD*, *rfaD* complemented strain by transformation with *pWF208*; Am^r (Ampicillin resistant)	This Study
HER1266	*E. coli* O157:H7 isolated from human stool	(Yu et al., 2000)
YQ254	HER1266:*ΔrfaD*, HER1266 isogenic mutant with *rfaD* deletion	This Study
YQ343	HER1266:Δ*rfaD-prfaD*, *rfaD* complemented strain by transformation with *pWF208*; Am^r	This Study
YQ368	OP50-GFP*, E. coli* OP50 transformed with pFPV25.1 plasmid; Am^r	(Chou et al., 2013)
YQ369	EDL933-GFP*, E. coli* O157:H7 EDL933 transformed with pFPV25.1 GFP plasmid; Am^r	(Chou et al., 2013)
YQ228	YQ217 transformed with pFPV25.1 GFP plasmid; Am^r	This Study
YQ275	YQ222 transformed with pWF264 GFP plasmid; Am^r and Kan^r	This Study
YQ306	EDL933 transformed with pWF279 luciferase plasmid; Kan^r	This Study
YQ307	YQ217 transformed with pWF279 luciferase plasmid; Kan^r	This Study
YQ308	YQ222 transformed with pWF279 luciferase plasmid; Am^r and Kan^r	This Study

Table S2. Plasmids used in this study

Plasmid	Description	Reference
pKD4	Template plasmid for Kan^r cassette	(Datsenko and Wanner, 2000)
pKD46	Expression of Red recombinase for deletion mutants construction; Am^r	(Datsenko and Wanner, 2000)
pWF208	*prfaD*, the RfaD expressing plasmid; Am^r	This Study
pFPV25.1	Vector for constitutive GFP expression; *rpsM*::*gfpmut;* Am^r	(Raphael H. Valdivia and Falkow, 1996)
pWF264	Vector for constitutive GFP expression; modified from pFVP25.1; Kan^r	This Study
pWF279	Luciferase expression plasmid; modified from pAKlux2 (Karsi and Lawrence, 2007), Kan^r	This Study

Table S3. Minimum inhibitory concentration (MIC) of Polymyxin B and Colistin

	MIC (µg/ml)	
Strains	Polymyxin B	Colistin
EDL933	1.33±0.28	1.50±0.27
ΔrfaD::Tn5	0.50±0.43*	0.25±0.00*
ΔrfaD	0.42±0.14*	0.25±0.00*
ΔrfaD-prfaD	1.33±0.28	1.17±0.26
E. coli ATCC 25922#	1.67±0.58	1.33±0.58

* All $P<0.05$ compared to EDL933 wild type.

\# *E. coli* quality control (QC) strain for Polymyxin B and Colistin (Wiegand et al., 2008).

References

Brenner, S. (1974). The genetics of Caenorhabditis elegans. *Genetics* 77(1), 71-94.

Chou, T.C., Chiu, H.C., Kuo, C.J., Wu, C.M., Syu, W.J., Chiu, W.T., et al. (2013). Enterohaemorrhagic Escherichia coli O157:H7 Shiga-like toxin 1 is required for full pathogenicity and activation of the p38 mitogen-activated protein kinase pathway in Caenorhabditis elegans. *Cell Microbiol* 15(1), 82-97. doi: 10.1111/cmi.12030.

Datsenko, K.A., and Wanner, B.L. (2000). One-step inactivation of chromosomal genes in Escherichia coli K-12 using PCR products. *Proc Natl Acad Sci U S A* 97(12), 6640-6645. doi: 10.1073/pnas.120163297.

Karsi, A., and Lawrence, M.L. (2007). Broad host range fluorescence and bioluminescence expression vectors for Gram-negative bacteria. *Plasmid* 57(3), 286-295. doi: 10.1016/j.plasmid.2006.11.002.

Raphael H. Valdivia, and Falkow, S. (1996). Bacterial genetics by flow cytometry: rapid isolation of Salmonella typhimurium acid-inducible promoters by differential fluorescence induction. *Molecular Microbiology* 22, 367-378.

Strockbine, N.A., Marques, L.R., Newland, J.W., Smith, H.W., Holmes, R.K., and O'Brien, A.D. (1986). Two toxin-converting phages from Escherichia coli O157:H7 strain 933 encode antigenically distinct toxins with similar biologic activities. *Infect. Immun.* 53.

Wiegand, I., Hilpert, K., and Hancock, R.E. (2008). Agar and broth dilution methods to determine the minimal inhibitory concentration (MIC) of antimicrobial substances. *Nat Protoc* 3(2), 163-175. doi: 10.1038/nprot.2007.521.

Yu, S.-L., Ko, K.-L., Chen, C.-S., Chang, Y.-C., and Syu, W.-J. (2000). Characterization of the Distal Tail Fiber Locus and Determination of the Receptor for Phage AR1, Which Specifically InfectsEscherichia coli O157:H7. *J. Bacteriol.* 182, 5962-5968.
